# Supplementary material for: Ryanodine receptor dispersion disrupts Ca2+ release in failing cardiac myocytes
Source: eLife. 2018 Oct 30;7:e39427. doi: 10.7554/eLife.39427 (PMC6245731; doi:10.7554/eLife.39427)
Supplement: Figure 2—source data 1. — RyR cluster and CRU data were compared in Sham and HF myocytes, with data cumulated across cells or animals. Two CRU definitions were also compared, with maximum edge-to-edge distances of 150 nm or 100 nm. Significant differences within data cumulated across cells were determined by t-test, while data cumulated across animals were tested with linear mixed effects models (Lindstrom and Bates, 1988). [file elife-39427-fig2-data1.pdf]

**Figure 2-source data 1**

| | | Cumulated Within Cells | | | Cumulated Within Animals | | |
|---|---|---|---|---|---|---|---|
| | | SHAM (n=46) | HF (n=50) | *P* | SHAM (n=6 ) | HF (n= 9) | *P* |
| 150 nm CRU | Clusters / µm$^2$ | 4.5 ± 0.3 | 6.4 ± 0.3 | <0.001 | 4.3 ± 0.6 | 6.3± 0.5 | 0.011 |
| 150 nm CRU | RyRs / Cluster | 13.7 ± 0.7 | 9.1 ± 0.4 | <0.001 | 14.7 ± 1.8 | 9.1 ± 0.8 | 0.002 |
| 150 nm CRU | RyRs / CRU | 34.1 ± 1.3 | 23.8 ± 0.9 | <0.001 | 34.3 ± 2.2 | 23.8 ± 1.5 | <0.001 |
| 150 nm CRU | Clusters / CRU | 3.7 ± 0.2 | 4.2 ± 0.2 | 0.049 | 3.1 ± 0.6 | 4.0 ± 0.4 | 0.175 |
| 150 nm CRU | Inter-cluster distance (nm) | 203 ± 8 | 170 ± 5 | <0.001 | 215 ± 20 | 170 ± 10 | 0.031 |
| 150 nm CRU | CRU solidity | 0.57 ± 0.01 | 0.52 ± 0.01 | 0.017 | 0.59 ± 0.03 | 0.52 ± 0.02 | 0.105 |
| 100 nm CRU | RyRs / CRU | 26.2 ± 1.0 | 17.2 ± 0.7 | <0.001 | 26.4 ± 2.0 | 17.3 ± 1.3 | <0.001 |
| 100 nm CRU | Clusters / CRU | 2.8 ± 0.1 | 2.9 ± 0.1 | 0.393 | 2.6 ± 0.3 | 3.0 ± 0.2 | 0.343 |
| 100 nm CRU | Inter-cluster distance (nm) | 215 ± 4 | 179 ± 6 | 0.002 | 228 ± 22 | 178 ± 12 | 0.028 |
| 100 nm CRU | CRU solidity | 0.63 ± 0.01 | 0.62 ± 0.01 | 0.365 | 0.66 ± 0.03 | 0.62 ± 0.02 | 0.301 |
