# Supplementary material for: Ryanodine receptor dispersion disrupts Ca2+ release in failing cardiac myocytes
Source: eLife. 2018 Oct 30;7:e39427. doi: 10.7554/eLife.39427 (PMC6245731; doi:10.7554/eLife.39427)
Supplement: Figure 4—source data 1. [file elife-39427-fig4-data1.pdf]

Figure 4-source data 1

	1 Cluster	3 Clusters	7 Clusters	10 Clusters
# RyRs	54	42	61	70
jSR volume (10^{-3} μm^3)	3.8	4.4	6.3	8.4
jSR volume / # RyRs	0.07	0.11	0.1	0.12
