# Supplementary figures and images for: Ryanodine receptor dispersion disrupts Ca2+ release in failing cardiac myocytes
Source: eLife. 2018 Oct 30;7:e39427. doi: 10.7554/eLife.39427 (PMC6245731; doi:10.7554/eLife.39427)

Figure 6-source data 1

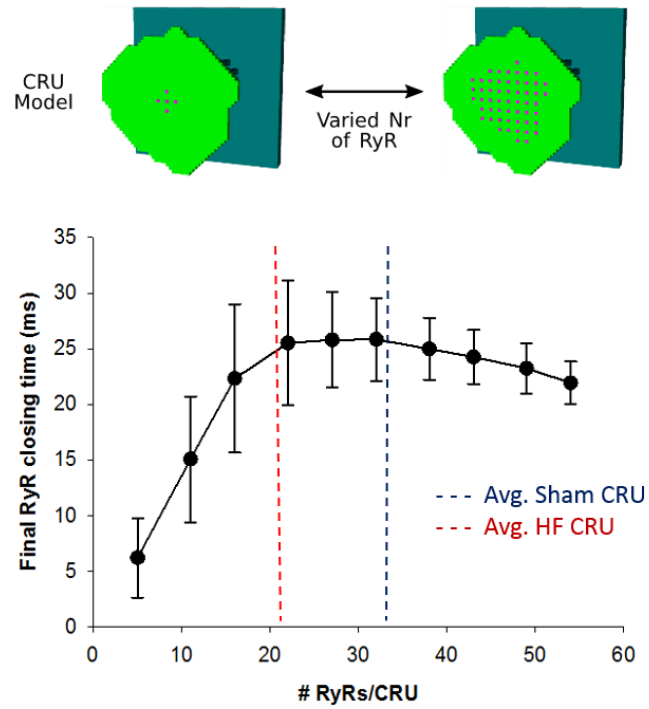

Supplement: Figure 6—source data 1. — Clusters smaller than 15 – 20 RyR exhibit early spark termination due to stochastic attrition, whereas larger clusters are more capable of supporting regenerative release, for which spark termination becomes relatively consistent in time and dependent on depletion of the releasable Ca2+ store. The simulations were started with all RyRs in the open state and allowed to proceed stochastically. For each RyR number, 50 simulations were performed. The plot shows the mean time from the start of the simulation until all RyRs close. Error bars are standard deviations. [file elife-39427-fig6-data1.pdf]
