# Supplementary material for: Ryanodine receptor dispersion disrupts Ca2+ release in failing cardiac myocytes
Source: eLife. 2018 Oct 30;7:e39427. doi: 10.7554/eLife.39427 (PMC6245731; doi:10.7554/eLife.39427)
Supplement: Supplementary file 1. [file elife-39427-supp1.docx]

**Supplementary file 1:**

|  | no. | $\sigma$( µm$^{2}/s$) | $B_{ tot }$ | $k_{ on }$(msµM) | $k_{ off }$(ms) |
| --- | --- | --- | --- | --- | --- |
| Calmodulin | 1 | 22 | 24 µM | 0.034 | 0.238 |
| ATP | 2 | 140 | 455 µM | 0.255 | 45 |
| Fluo-4 | 3 | 42 | 25 µM | 1.1 | 1.1 |
| Troponin | 4 | 0 | 70 µM | 0.0327 | 0.0196 |
| Calsequestrin | 5 | 0 | 30 mM | 0.102 | 65 |
