# Supplementary material for: Ryanodine receptor dispersion disrupts Ca2+ release in failing cardiac myocytes
Source: eLife. 2018 Oct 30;7:e39427. doi: 10.7554/eLife.39427 (PMC6245731; doi:10.7554/eLife.39427)
Supplement: Supplementary file 2. [file elife-39427-supp2.docx]

**Supplementary file 2:**

|  | $k_{min}$ (ms^-1^) | $k_{max}$(ms^-1^) | $K$ (µM) | $n$ |
| --- | --- | --- | --- | --- |
| + | 5 x 10^-6^ | 0.7 | 80 | 2.8 |
| - | 0.9 | 3 | 62.5 | -0.5 |
